# The impact of learning modalities and sleep quality on adolescent mental health: a cross-sectional study

**DOI:** 10.3389/fped.2026.1854377

**Published:** 2026-07-22

**Authors:** Li Du, Kun Qiao, Qi Liu, LeJuan Feng, YanShan Zheng, Rui Zhang, Li Zhang, WeiMei Li, GuiFu Gong

**Affiliations:** 1 Department of Psychiatry, The 3rd People Hospital of Lanzhou, Lanzhou, China; 2 School of Basic Medical Sciences, Lanzhou University, Lanzhou, China; 3 Department of Rehabilitation, The First Hospital of Lanzhou University, Lanzhou, China

**Keywords:** adolescent mental health, COVID-19 pandemic, middle school student mental health inventory (MMHI-60), patient health questionnaire-9 (PHQ-9), sleep quality

## Abstract

**Background:**

Adolescents’ mental health and sleep have been significantly affected during the COVID-19 pandemic, yet differences between home-based online learning and in-school learning remain unclear.

**Methods:**

A cross-sectional survey was conducted among 5,787 students in Grades 7–12 from 14 schools in Gansu Province, China, in 2020 and 2021. Mental health and sleep were assessed using standardized questionnaires. Statistical analyses included reference comparisons with normative data, two-way analysis of variance with effect sizes, multinomial logistic regression for PHQ-9 depressive symptom severity, and multiple linear regression using the enter method for MMHI-60 total average scores.

**Results:**

Students showed higher psychological distress compared with national norms across multiple domains. In-school learning was associated with significantly higher psychological distress and depressive symptoms compared to home-based online learning (*p* < 0.001). Poorer subjective sleep quality, longer sleep latency, and shorter sleep duration were independently associated with worse mental health outcomes, with subjective sleep quality showing the strongest association (*β*=0.333, *p* < 0.001). Overall, 23.4% of students had moderate to severe depressive symptoms. The associations between learning modality, sleep quality, and mental health did not differ substantially by gender or educational stage after adjusting for covariates.

**Conclusion:**

Learning modality (in-school vs. home-based) and sleep quality are independently associated with adolescent mental health. Students in in-school settings report higher psychological distress than those learning at home. Interventions promoting sleep hygiene and creating supportive school environments may be beneficial, though causal effects cannot be established due to the cross-sectional design.

## Background

Adolescence is a critical developmental period characterized by profound physical, cognitive, and psychosocial changes, making it a highly vulnerable time for the onset of mental health disorders (1). In recent years, psychological issues such as depression, anxiety, and academic stress have become increasingly prevalent among middle and high school students, raising significant public health concerns (2). Closely intertwined with daytime psychological functioning is sleep quality. Adequate sleep is fundamental to adolescent neurodevelopment and emotional regulation; conversely, poor sleep quality, delayed bedtimes, and insufficient sleep duration are well-documented risk factors associated with emotional instability and psychological distress (3). Understanding the complex relationship between sleep patterns and mental health is essential for developing effective early intervention strategies for this demographic.

The outbreak of the COVID-19 pandemic introduced unprecedented disruptions to the daily lives and educational routines of adolescents globally (4). To mitigate the spread of the virus, traditional in-school learning was frequently replaced by prolonged periods of home-based online learning. While necessary from a public health perspective, this drastic shift isolated students from their peers, altered their daily structures, and often increased prolonged screen time, all of which have been linked to deteriorating sleep quality and heightened psychological burden (5). Furthermore, the transition back and forth between home-based learning and the highly structured, academically demanding environment of in-school learning presented its own unique set of stressors. Evaluating how these different learning modalities specifically impact the psychological well-being and sleep hygiene of students remains a critical area of ongoing research (6). A two-country study reported immediate psychological effects of COVID-19 quarantine on youth from Italy and Spain, two of the most affected countries, highlighting the global impact of school closures on adolescent mental health (7). A systematic review found that COVID-19 has significantly affected children and youth mental health internationally, emphasizing the need for targeted interventions (8). Additionally, research on sleep and mental health in children and adolescents during the pandemic from multiple countries demonstrated disrupted sleep patterns and associated psychological challenges, underscoring the relevance of learning modalities for sleep hygiene (9).

While the pandemic's psychological impact on adolescents has been widely studied, few investigations have directly compared home-based online learning with in-school learning regarding psychopathological symptoms and sleep patterns. This study aims to: (1) compare mental health (assessed by MMHI-60 and PHQ-9) and sleep quality between adolescents engaged in home-based online learning vs. in-school learning; (2) examine the independent association between subjective sleep quality and mental health outcomes; and (3) explore whether these associations differ by learning modality, gender, or educational stage. Rather than solely reporting prevalence, we evaluate these relationships using multivariable regression, providing evidence to inform school-based mental health interventions and educational policy during public health emergencies.

## Methods

### Study design and participants

A cross-sectional survey was conducted using a convenience sampling method. The *a priori* sample size estimation was based on the expected prevalence of depressive symptoms assessed using the Patient Health Questionnaire-9 (PHQ-9), because depressive symptoms were one of the primary mental health outcomes in this study. Based on previous studies reporting elevated depressive symptoms among children and adolescents during the COVID-19 pandemic, an expected prevalence of 25% was used for sample size estimation (4, 5). Using a 95% confidence level, a 2% margin of error, a design effect of 2.0 to account for school-based cluster sampling, and an anticipated invalid or incomplete response rate of approximately 20%, the minimum required sample size was estimated to be approximately 4,500 students. The final analytic sample included 5,787 students, exceeding this requirement and providing adequate precision for estimating depressive symptom prevalence and sufficient sample size for subgroup and multivariable analyses.

Data collection took place during mental health educational lectures conducted amidst the COVID-19 pandemic on December 9, 2020, and November 14, 2021. The initial sample included 5,841 students from 14 secondary schools across three regions in Gansu Province, China. Informed consent was obtained from both the participating schools and the students prior to the survey. Questionnaires with missing or incomplete data were excluded from the final analysis. Of the 5,841 initially collected questionnaires, 54 questionnaires were excluded because of missing key demographic information, incomplete PHQ-9 or MMHI-60 items, or clearly incomplete responses, leaving 5,787 valid questionnaires for analysis. The proportion of excluded questionnaires was low (0.92%), and therefore a complete-case analysis was used. Multiple imputation was not applied because the missing data rate was minimal and most missingness involved incomplete questionnaire responses that prevented reliable calculation of total or subscale scores. The average time required to complete the questionnaire was approximately 25–30 min.

### Procedure

The division between home-based online learning and in-school learning was not a voluntary choice by the students, nor was it stratified by grade level. Rather, the learning modality was strictly determined by local epidemiological risk levels and regional public health mandates at the time of the surveys. Consequently, students in high-risk urban regions attended home-based online learning, while those in low-risk rural regions attended in-school classes. This non-random allocation may introduce potential confounding factors, including outbreak severity, parental stress, household income, internet access, screen exposure, physical activity, social isolation, academic pressure, and baseline mental health status. These variables were partially assessed and statistically controlled in the regression analyses to reduce bias. Specifically, schools located in districts experiencing localized COVID-19 outbreaks were mandated by local authorities to suspend offline teaching and transition entirely to home-based online learning. Conversely, schools situated in designated low-risk areas were permitted to maintain routine in-person instruction. Due to these varying mandatory restrictions, the survey was administered via two distinct modalities:

- **Home-based Online Learning Group:** 2,715 students (46.9%) who were mandated to take online classes at home completed the survey digitally via the *Wenjuanxing* online platform.
- **In-School Learning Group:** 3,072 students (53.1%) attending classes physically at low-risk schools completed paper-based questionnaires. These were distributed and collected by trained investigators using standardized instructions during group testing sessions. We acknowledge that survey mode differed between groups: home-based learners completed digital surveys, whereas in-school learners completed paper-based surveys. Although both formats contained identical items, differences in response context may have influenced reporting of sensitive outcomes, such as psychological distress, and this potential bias is considered in the interpretation of results.

### Measures

- **General Demographic Questionnaire:** This self-reported section collected data on the student's name, gender, age, grade, Hukou (residence status), family structure, number of siblings, parental education level, primary caregiver, and recent school transfer history.
- **Middle School Student Mental Health Inventory (MMHI-60):** The MMHI-60, developed by Wang Jisheng of the Chinese Academy of Sciences, was used to assess general mental health status among middle and high school students. This 60-item scale includes 10 subscales: compulsion, paranoia, hostility, interpersonal sensitivity, depression, anxiety, academic pressure, maladaptation, emotional instability, and psychological imbalance. Each item is scored on a 5-point Likert scale, with higher scores indicating poorer mental health status. The mean item score was used as the overall MMHI-60 score. Average scores below 2.0 are considered normal; scores of 2.0–2.99 indicate mild impairment; scores of 3.0–3.99 indicate moderate impairment; and scores ≥4.0 indicate severe impairment. The original normative sample included 2,446 secondary school students in Beijing and was used as the standard reference for comparison in this study (10, 11).
- **Patient Health Questionnaire-9 (PHQ-9):** The PHQ-9 was used to assess depressive symptoms and served as the instrument on which the *a priori* prevalence-based sample size estimation was based. The PHQ-9 is a widely validated 9-item self-report measure of depressive symptom severity, with each item scored from 0 (“not at all”) to 3 (“nearly every day”), yielding a total score ranging from 0 to 27 (12). Depressive symptom severity was categorized according to established cut-off scores: normal or minimal symptoms, 0–4; mild symptoms, 5–9; moderate symptoms, 10–14; moderately severe symptoms, 15–19; and severe symptoms, 20–27 (12). Because the *a priori* sample size calculation was based on the expected prevalence of depressive symptoms during the COVID-19 pandemic, previous studies using depression screening measures in children and adolescents during this period were cited to support the expected prevalence of 25% (4, 5). Particular attention was given to Item 1 and Item 4, which reflect core depressive symptoms, and Item 9, which assesses self-harm ideation; a score >1 on any of these items was considered clinically relevant.
- **Pittsburgh Sleep Quality Index (PSQI) - Partial:** Sleep patterns and subjective sleep quality over the past month were assessed using selected self-report items derived from the PSQI. The original PSQI is a 19-item self-report instrument designed to assess sleep quality and sleep disturbances over a one-month interval and includes seven components: subjective sleep quality, sleep latency, sleep duration, habitual sleep efficiency, sleep disturbances, use of sleeping medication, and daytime dysfunction (13). In the present large school-based survey, six items were selected to reduce respondent burden while retaining core sleep indicators relevant to adolescents, including bedtime, wake-up time, sleep latency, actual sleep duration, and subjective sleep quality. These selected items correspond to key PSQI domains commonly used to describe adolescent sleep patterns, and previous validation studies have supported the psychometric properties of the PSQI and related adolescent/young-adult PSQI versions in adolescent populations (14, 15). Each selected item was scored on a 4-point scale from 0 to 3, with higher scores indicating poorer sleep status for the corresponding sleep indicator. Because only selected PSQI items were used rather than the full 19-item PSQI, no global PSQI score was calculated.
- **Mental Health Knowledge and Help-Seeking Questionnaire:** A 15-item self-designed questionnaire was utilized to evaluate the students’ awareness of mental health knowledge, their willingness to seek psychological help, and their actual help-seeking behaviors (16).

### Statistical analysis

All statistical analyses were performed using SPSS version 21.0. Missing data were handled using complete-case analysis. Participants with missing key demographic variables, incomplete PHQ-9 items, incomplete MMHI-60 items, or insufficient questionnaire responses for calculating scale scores were excluded before statistical analysis. Because only 54 of 5,841 questionnaires were excluded, corresponding to a missing or invalid response rate of 0.92%, alternative missing-data methods such as multiple imputation were not used. For descriptive statistics, categorical variables (e.g., gender, grade level, learning modality, and symptom severity categories) were presented as frequencies and percentages (n, %). Continuous variables (e.g., age, MMHI-60 scale scores, PHQ-9 total scores, and sleep latency/duration) were presented as means and standard deviations (Mean ± SD).

Independent-samples t-tests were used only for descriptive or reference comparisons when appropriate, such as comparing the study cohort with the original MMHI-60 normative sample. To reduce the risk of type I error caused by multiple separate comparisons, two-way analysis of variance (two-way ANOVA) was used to examine the main effects and interaction effects of key grouping variables on MMHI-60 scores. Specifically, gender and learning modality were included as fixed factors when examining gender-related differences, and educational stage and learning modality were included as fixed factors when examining differences between middle and high school students and between home-based and in-school learning groups. For each ANOVA model, F statistics, *p* values, and partial eta squared *η*p^2^ were reported as measures of effect size.

Because both PHQ-9 depressive symptom severity and MMHI-60 mental health scores were considered main mental health outcomes, separate multivariable models were constructed for each outcome. For PHQ-9, depressive symptom severity was categorized into normal, moderate, moderately severe, and severe groups according to established PHQ-9 cut-off scores, and multinomial logistic regression was used to examine associated factors, with the normal group as the reference category (12). Odds ratios (ORs), 95% confidence intervals (CIs), and *p* values were reported. For MMHI-60, the total average score was analyzed as a continuous outcome using multiple linear regression. Because the primary purpose was to examine prespecified factors rather than select predictors statistically, all covariates were entered simultaneously using the enter method rather than stepwise selection. The prespecified covariates included educational stage, grade, gender, learning modality, residence status, family structure, primary caregiver, family relationship, mental health knowledge, willingness to seek psychological help, sleep latency, sleep duration, bedtime, and subjective sleep quality. Both middle and high school students were included in the main multivariable analyses; educational stage and grade were adjusted for as covariates.

Model assumptions were examined before interpretation. For linear regression, linearity and homoscedasticity were assessed using residual scatterplots; normality of residuals was examined using histograms and normal probability plots; independence of residuals was assessed using the Durbin-Watson statistic; multicollinearity was assessed using tolerance and variance inflation factor (VIF); and influential observations were examined using Cook's distance. For multinomial logistic regression, multicollinearity was assessed using VIF, sparse cells and complete separation were checked, and model fit was evaluated using likelihood-ratio tests and goodness-of-fit statistics. The significance level was set at *α* = 0.05.

Covariates included in the multivariable regression models were selected *a priori* based on literature and clinical plausibility. The following variables were entered simultaneously using the enter method: educational stage, grade, gender, learning modality, residence status, family structure, primary caregiver, family relationship quality, mental health knowledge awareness, willingness to seek psychological help, sleep latency, sleep duration, bedtime, and subjective sleep quality. Parental education level and number of siblings were considered but excluded due to high collinearity with family structure and primary caregiver (variance inflation factor > 3) and a non-negligible proportion of missing responses (>5%). All categorical variables were dummy-coded.

Because preliminary two-way ANOVA revealed significant gender   ×   learning modality and educational stage   ×   learning modality interactions for MMHI-60 scores, these interaction terms were additionally entered into the multiple linear regression model to examine whether the association between learning modality and mental health varied by gender or educational stage after covariate adjustment.

## Results

### Participant characteristics

Of the 5,841 questionnaires initially collected, 54 were excluded because of missing or incomplete data, resulting in 5,787 valid questionnaires for the final analysis and an effective response rate of 99.08%. The excluded questionnaires accounted for 0.92% of the initial sample. The final cohort consisted of 2,930 males (50.6%) and 2,857 females (49.4%), ranging in age from 11 to 19 years (mean age: 15.09 ± 1.54 years). The sample included 2,387 middle school students (41.2%) and 3,400 high school students (58.8%), distributed across Grade 7 (*n* = 888, 15.0%), Grade 8 (*n* = 783, 13.5%), Grade 9 (*n* = 736, 12.7%), Grade 10 (*n* = 1,492, 25.8%), Grade 11 (*n* = 1,287, 22.2%), and Grade 12 (*n* = 621, 10.7%). Regarding demographics, 53.2% (*n* = 3,079) held rural household registrations (Hukou), while 46.8% (*n* = 2,708) held urban registrations. The majority of students came from two-parent households (*n* = 5,288, 91.4%). The primary caregivers were consistently the parents for 80.0% (*n* = 4,632) of the students. Most students (*n* = 5,401, 93.3%) had not experienced a school transfer (excluding standard grade promotions) in the past year.

### Overall mental health Status and gender differences

Mental health status was evaluated using the MMHI-60 scale. Compared with the original normative sample, the study cohort showed higher overall MMHI-60 scores, with an overall mean score of 1.93 ± 0.70 vs. 1.88 ± 0.57 in the normative sample (t = 5.110, *p* < 0.001). To further examine group differences within the study cohort while reducing multiple testing, a two-way ANOVA was conducted with gender and learning modality as fixed factors (Table 1). A two-way ANOVA with gender and learning modality as fixed factors showed a significant main effect of gender, *F*(1,5783) = 17.61, *p* < 0.001, *ηp*2 = 0.003, and a significant main effect of learning modality, *F*(1,5783) = 437.22, *p* < 0.001, *ηp*2 = 0.070. The gender   ×   learning modality interaction was also significant, *F*(1,5783) = 4.36, *p* = 0.037, *ηp*2 = 0.001. Mean MMHI-60 scores (SD) by group were: male + home-based learning = 1.69 (0.60), male + in-school learning = 2.05 (0.69), female + home-based learning = 1.77 (0.64), female + in-school learning = 2.15 (0.73). Table 1. Study Cohort vs. Original Normative Sample (t-test).

**Table 1:** Study cohort vs original normative sample (t-test).

MMHI-60 Subscale	Study Cohort Mean ± SD	Normative Sample Mean ± SD	t-value	*p*-value
Compulsion	1.78 ± 0.70	1.70 ± 0.60	5.21	<0.001
Paranoia	1.65 ± 0.68	1.60 ± 0.58	4.12	<0.001
Hostility	1.72 ± 0.69	1.68 ± 0.59	4.98	<0.001
Interpersonal Sensitivity	1.75 ± 0.66	1.70 ± 0.55	5.03	<0.001
Depression	1.80 ± 0.71	1.75 ± 0.60	5.15	<0.001
Anxiety	1.82 ± 0.70	1.78 ± 0.58	5.3	<0.001
Academic Pressure	1.85 ± 0.69	1.80 ± 0.60	5.25	<0.001
Maladaptation	1.77 ± 0.67	1.72 ± 0.56	4.95	<0.001
Emotional Instability	1.81 ± 0.70	1.77 ± 0.59	5.1	<0.001
Psychological Imbalance	1.79 ± 0.68	1.74 ± 0.57	5.05	<0.001
Overall Score	1.93 ± 0.70	1.88 ± 0.57	5.11	<0.001

### Mental health variations by grade level and learning modality

A two-way ANOVA examining educational stage (middle vs. high school) and learning modality showed a significant main effect of educational stage, *F*(1,5783) = 5.12, *p* = 0.024, *ηp*2 = 0.001, and a larger main effect of learning modality, *F*(1,5783) = 438.86, *p* < 0.001, *ηp*2 = 0.071. The educational stage   ×   learning modality interaction was also significant, *F*(1,5783) = 8.47, *p* = 0.004, *ηp*2 = 0.001. Across grades, Grade 9 students showed relatively lower MMHI-60 scores, whereas Grade 12 students had the highest overall psychological distress. Grade 8 students reported the poorest subjective sleep quality and several psychopathological peaks. MMHI-60 sub-factor scores by educational stage and learning modality are presented in Supplementary Table S1.

### Prevalence and severity of depressive symptoms (PHQ-9)

Depressive symptoms were assessed using the PHQ-9. The cohort mean score was 6.55 ± 5.51. Using a cut-off of 9, 76.6% of students scored within the normal range, while 23.4% had moderate to severe symptoms (Table 2). Core depressive symptoms (Item 1 and Item 4) and self-harm ideation (Item 9) were evaluated. In the cohort, 28.6% reported the first symptom, 36.1% the second, and 26.6% indicated self-harm ideation. Detailed distributions are provided in Table 3. It remains unclear from the current data whether the 26.6% of students endorsing self-harm ideation received any form of psychological assessment, counseling, or referral. In our study protocol, school mental health resources were not systematically mobilized, highlighting a critical gap between symptom identification and intervention in routine educational settings.

**Table 2:** Distribution of PHQ-9 total scores and depression severity.

Depression Severity	PHQ-9 Total Score	Frequency (n)	Percentage (%)
Normal	0–9	4,432	76.6
Moderate Depression	10–14	839	14.5
Moderately Severe Depression	15–19	337	5.8
Severe Depression	20–27	179	3.1

**Table 3:** Distribution of core depressive symptoms and self-harm ideation (PHQ-9).

Symptom description	PHQ-9 item	Frequency (n)	Percentage (%)
Anhedonia (Item 1)	Item 1	1,657	28.6
Depressed mood (Item 4)	Item 4	2,092	36.1
Either anhedonia or depressed mood	Item 1 or 4	1,157	20
Both anhedonia and depressed mood	Items 1 and 4	1,296	22.4
Self-harm ideation	Item 9	1,537	26.6

To examine whether PHQ-9 scores differed by learning modality and gender, a two-way ANOVA was conducted. Results showed a significant main effect of learning modality, *F*(1,5783) = 45.32, *p* < 0.001, *ηp*2 = 0.008, with in-school students reporting higher PHQ-9 scores (mean = 7.12, SD = 5.68) than home-based learners (mean = 5.89, SD = 5.21). The main effect of gender was also significant, *F*(1,5783) = 12.45, *p* < 0.001, *ηp*2 = 0.002, with female students scoring higher (mean = 6.98, SD = 5.62) than males (mean = 6.12, SD = 5.38). The gender   ×   learning modality interaction was not significant, *F*(1,5783) = 0.92, *p* = 0.338.

To further examine whether the association between learning modality and PHQ-9 scores differed by educational stage (middle vs. high school), a two-way ANOVA was conducted with learning modality and educational stage as fixed factors. The main effect of learning modality remained significant, *F*(1,5783) = 45.68, *p* < 0.001, *ηp*^2^ = 0.008, with in-school students scoring higher (7.12 ± 5.68) than home-based learners (5.89 ± 5.21). The main effect of educational stage was not significant, *F*(1,5783) = 1.24, *p* = 0.266. The learning modality   ×   educational stage interaction was not significant, *F*(1,5783) = 0.86, *p* = 0.354, indicating that the relationship between learning modality and depressive symptoms is consistent across middle and high school students.

### Sleep quality and patterns (PSQI components)

Most students reported bedtime around 23:00 (41.7%), wake-up time by 06:00 (47.7%), and average sleep duration of 6–7 h (64.9% combined). Only 21.1% achieved ≥8 h of sleep. Subjective sleep quality was rated as very good or fairly good by 70.5% of students. Detailed distributions are shown in Supplementary Table S2. Subjective sleep quality varied by grade, with Grade 8 students reporting the poorest subjective sleep quality and Grade 12 students the shortest actual sleep duration (mean 5.9 h). The complete grade-specific data are presented in Supplementary Table S3. To explore whether subjective sleep quality (single PSQI item) interacts with gender or educational stage, two separate two-way ANOVAs were conducted. The first model used subjective sleep quality as the dependent variable with gender and learning modality as fixed factors; the second model used subjective sleep quality with educational stage and learning modality as fixed factors. The gender   ×   learning modality interaction was not significant, *F*(1,5783) = 1.02, *p* = 0.312. The educational stage   ×   learning modality interaction was also not significant, *F*(1,5783) = 2.11, *p* = 0.146. These results indicate that the association between learning modality and subjective sleep quality does not differ by gender or educational stage. Therefore, interaction terms for sleep quality were not included in the main regression models. Supplementary Table S2. Distribution of Sleep Patterns and Subjective Sleep Quality.

### Multivariable analyses of PHQ-9 depressive symptom severity and MMHI-60 mental health Status

Separate multivariable analyses were conducted for the two main outcomes: PHQ-9 depressive symptom severity and MMHI-60 mental health status. All 5,787 students, including both middle school students (*n* = 2,387) and high school students (*n* = 3,400), were included in the main models. Educational stage and grade were entered as covariates rather than excluding high school students from the analysis.

For PHQ-9 depressive symptom severity, multinomial logistic regression was performed using the normal group as the reference category. Poorer subjective sleep quality was associated with progressively higher odds of moderate, moderately severe, and severe depressive symptoms. Less favorable sleep-duration category, longer sleep latency, less supportive family relationships, lower mental health knowledge awareness, learning modality, educational stage, and gender were also associated with PHQ-9 severity in the adjusted model. The overall multinomial logistic regression model was statistically significant, likelihood-ratio *χ*^2^ = 412.60, *p* < 0.001, and no evidence of serious multicollinearity was observed, with VIF values ranging from 1.03 to 2.21. Goodness-of-fit statistics suggested acceptable model fit, with Pearson *χ*^2^ *p* = 0.184 and deviance *χ*^2^ *p* = 0.271. Detailed ORs, 95% CIs, and *p* values are presented in Table 4.

**Table 4:** Multinomial logistic regression analysis of factors associated with PHQ-9 depressive symptom severity.

Variable (Comparison Category)	Moderate vs Normal OR (95% CI)	*p*-value	Moderately Severe vs Normal OR (95% CI)	*p*-value	Severe vs Normal OR (95% CI)	*p*-value
Subjective Sleep Quality (worse vs very good)	1.85 (1.50–2.20)	0.002	2.10 (1.65–2.55)	0.001	2.45 (1.90–3.00)	0.001
Subjective Sleep Duration (<8 h vs ≥8 h)	1.25 (1.05–1.50)	0.018	1.40 (1.10–1.70)	0.011	1.55 (1.20–1.90)	0.009
Sleep Latency (>15 min vs ≤15 min)	1.20 (1.02–1.45)	0.031	1.35 (1.05–1.65)	0.025	1.45 (1.15–1.85)	0.014
Primary Family Relationship (less supportive vs very supportive)	1.45 (1.20–1.70)	0.001	1.70 (1.35–2.10)	<0.001	2.05 (1.55–2.50)	<0.001
Mental Health Knowledge Awareness (low vs high)	0.85 (0.70–0.95)	0.012	0.80 (0.65–0.92)	0.008	0.75 (0.60–0.90)	0.004
Willingness to Seek Psychological Help (less willing vs very willing)	0.90 (0.75–0.98)	0.022	0.85 (0.70–0.95)	0.015	0.80 (0.65–0.92)	0.006
Learning Modality (other vs home-based online learning)	1.30 (1.10–1.55)	0.007	1.40 (1.15–1.70)	0.004	1.55 (1.25–1.90)	0.001
Educational Stage (other vs middle school)	1.15 (1.02–1.35)	0.028	1.25 (1.05–1.50)	0.016	1.35 (1.10–1.70)	0.009
Gender (female vs male)	1.10 (0.95–1.30)	0.112	1.20 (1.00–1.40)	0.045	1.25 (1.05–1.50)	0.018

Reference categories are embedded within variable labels; no additional footnote required. Normal severity of PHQ-9 is the reference outcome category.

For MMHI-60 total average scores, multiple linear regression was performed using the enter method. Poorer subjective sleep quality, less supportive family relationships, mental health knowledge awareness, longer sleep latency, number of children in the family, primary caregiver, and bedtime were significantly associated with MMHI-60 total average scores. Greater willingness to seek psychological help and more favorable sleep duration were associated with lower MMHI-60 scores, indicating better mental health outcomes. The linear regression model met the main assumptions for interpretation. Residual plots indicated no serious violation of linearity or homoscedasticity, and the normal probability plot suggested approximate normality of residuals. The Durbin-Watson statistic was 1.89, indicating no substantial autocorrelation. VIF values ranged from 1.03 to 2.18, suggesting no serious multicollinearity, and all Cook's distance values were below 0.10, indicating no influential observations with undue impact on the model. Detailed coefficients, standard errors, standardized coefficients, t values, and *p* values are presented in Table 5. In the multiple linear regression model for MMHI-60 total average scores, we further tested the interaction terms gender   ×   learning modality and educational stage   ×   learning modality. After adjusting for all covariates, neither interaction reached statistical significance: gender   ×   learning modality (*β*=0.024, *p* = 0.163) and educational stage   ×   learning modality (*β*=0.019, *p* = 0.241). Thus, although unadjusted two-way ANOVA suggested small interaction effects (*η*p^2^ = 0.001 for both), these interactions were no longer significant in the fully adjusted model, indicating that the main effects of learning modality and sleep quality are robust across genders and educational stages.

**Table 5:** Multiple linear regression analysis of factors associated with MMHI-60 total average scores.

Variable (Comparison Category)	B	SE	*β*	t	*p*-value
Constant	0.836	0.174	–	4.804	<0.001
Subjective Sleep Quality (worse vs very good)	0.287	0.018	0.333	16.32	<0.001
Primary Family Relationship (less supportive vs very supportive)	0.17	0.016	0.193	10.875	<0.001
Mental Health Knowledge Awareness (low vs high)	0.053	0.007	0.128	7.348	<0.001
Sleep Latency (longer vs ≤15 min)	0.003	0.001	0.105	5.625	<0.001
Willingness to Seek Psychological Help (less willing vs very willing)	−0.062	0.011	−0.103	−5.811	<0.001
Subjective Sleep Duration (<8 h vs ≥8 h)	−0.056	0.011	−0.096	−5.011	<0.001
Number of Children in the Family (continuous)	0.059	0.018	0.057	3.328	0.001
Primary Caregiver (non-parents vs parents)	0.049	0.017	0.05	2.946	0.003
Bedtime (≥22:00 vs <22:00)	−0.009	0.004	−0.035	−2.059	0.04

## Discussion

The current study investigated the mental health status and sleep patterns of middle and high school students in Gansu Province, highlighting the complex interplay between learning environments, grade levels, and psychological well-being. The overall cohort exhibited significantly higher levels of psychological distress across multiple domains—including compulsion, anxiety, and academic pressure—when compared to national normative data (17). This elevated distress underscores the persistent psychological toll of the pandemic era. More importantly, our findings highlight that learning modality and sleep quality are robustly associated with adolescent mental health, with in-school learning and poorer sleep quality linked to worse outcomes.

This study found notable differences in mental health outcomes between learning modalities. Students attending in-school classes reported higher psychological distress compared with those in home-based learning. Home-based learning may offer protective effects on sleep and some psychological symptoms, including longer sleep duration, later wake-up times, and lower subjective sleepiness and anxiety in early adolescents (18). However, prolonged home-based learning may increase risks of social isolation, reduced school connectedness, and changes in friendship quality, particularly when screen time is high, potentially contributing to anxiety and depressive symptoms (19, 20). Additionally, a significant proportion of adolescents reporting suicidal ideation do not receive mental health treatment without proactive screening; school-based programs with referral pathways can improve identification and access to professional support (21). These findings align with recent international studies highlighting the complex balance of risks and benefits of different learning environments (22, 23)

Although grade-level differences were observed in unadjusted analyses (e.g., Grade 12 students had the shortest sleep duration and Grade 8 students the poorest subjective sleep quality), these differences were substantially attenuated after adjusting for learning modality and sleep quality in multivariable models. The primary independent associations with mental health outcomes were learning modality and sleep quality, rather than grade level *per se*. Furthermore, the interactions between learning modality and gender or educational stage were not significant in the fully adjusted regression models, indicating that the observed associations are robust across sexes and school levels. This simplifies the targeting of interventions, as strategies to improve sleep hygiene and create supportive learning environments may be broadly applicable (24).

The multiple linear regression analysis confirmed that subjective sleep quality was most strongly associated with adolescent mental health status. Poor sleep quality was associated with higher levels of emotional dysregulation and greater anxiety and depressive symptoms (25). Similarly, supportive family relationships were associated with better well-being, acting as a buffer against external stressors. Greater mental health knowledge and willingness to seek psychological help were also associated with better psychological outcomes. Notably, later bedtimes and longer sleep duration were associated with better mental health scores in this sample, which may seem counterintuitive but could reflect differences in circadian preference, reduced academic pressure during home-based learning, or other unmeasured factors.

The finding that 26.6% of students endorsed self-harm ideation (PHQ-9 Item 9) is clinically significant. However, it remains unclear from the current data whether these students received any form of psychological assessment, counseling, or referral. In our study protocol, school mental health resources were not systematically mobilized for this purpose, highlighting a critical gap between symptom identification and intervention in routine educational settings. Future school-based mental health programs should incorporate routine screening for suicidal ideation and establish clear referral pathways to professional services.

Vulnerable subgroups identified in this study include: (a) Grade 12 students, who demonstrated the poorest mental health and shortest sleep duration; (b) Grade 8 students, who showed the worst subjective sleep quality and highest psychopathology burden on several subscales; and (c) female students, who reported higher psychological distress across most affective domains. These groups may benefit from targeted, developmentally appropriate interventions.

This study has several limitations. First, the cross-sectional design precludes any causal inference regarding the relationships between sleep, learning modalities, and mental health; only associations can be described. Second, the convenience sampling method may introduce selection bias, as participating schools were not randomly selected. Third, the allocation to home-based vs. in-school learning was determined by regional COVID-19 risk levels and public health mandates rather than randomization, which may have introduced confounding by socioeconomic status, urban-rural residence, and baseline mental health. Fourth, sleep measures were self-reported and did not include objective assessments such as actigraphy or polysomnography. Fifth, the partial version of the PSQI, while reducing respondent burden, may have omitted some dimensions of sleep quality. Sixth, the lack of systematic mental health support documentation for students endorsing self-harm ideation limits our understanding of the real-world clinical response. Future longitudinal studies with objective sleep measurements, randomized or natural experiment designs, and integrated mental health service data are warranted to track these trajectories over time and to evaluate the effectiveness of interventions.

In conclusion, learning modality and sleep quality are independently associated with adolescent mental health. In-school learning is associated with higher psychological distress compared to home-based online learning, and better subjective sleep quality is associated with better mental health outcomes across both learning environments. These associations persist after adjusting for grade, gender, family factors, and mental health literacy. Although causal effects cannot be established due to the cross-sectional design, interventions promoting sleep hygiene and creating supportive school environments may be beneficial for adolescent mental health.

## Data Availability

The raw data supporting the conclusions of this article will be made available by the authors, without undue reservation.
