# Supplementary material for: The impact of learning modalities and sleep quality on adolescent mental health: a cross-sectional study
Source: Front Pediatr. 2026 Jul 22;14:1854377. doi: 10.3389/fped.2026.1854377 (PMC13437675; doi:10.3389/fped.2026.1854377)
Supplement: Supplementary file 1 [file Table1.docx]

Supplementary Table S1. MMHI-60 Sub-factor Scores by Educational Stage and Learning Modality

| **MMHI-60 Sub-factor** | **Middle School n = 2,387 Mean ± SD** | **High School n = 3,400 Mean ± SD** | **Home-based Learning n = 2,715 Mean ± SD** | **In-school Learning n = 3,072 Mean ± SD** |
| --- | --- | --- | --- | --- |
| Compulsion | 2.09 ± 0.72 | 2.05 ± 0.71 | 1.89 ± 0.68 | 2.22 ± 0.71 |
| Paranoia | 1.86 ± 0.80 | 1.77 ± 0.74 | 1.58 ± 0.64 | 2.01 ± 0.81 |
| Hostility | 1.90 ± 0.92 | 1.70 ± 0.76 | 1.58 ± 0.70 | 1.96 ± 0.90 |
| Interpersonal Sensitivity | 1.97 ± 0.85 | 1.92 ± 0.79 | 1.70 ± 0.70 | 2.15 ± 0.85 |
| Depression | 1.99 ± 0.90 | 1.92 ± 0.81 | 1.74 ± 0.75 | 2.14 ± 0.89 |
| Anxiety | 2.13 ± 1.01 | 2.05 ± 0.93 | 1.84 ± 0.87 | 2.29 ± 1.00 |
| Academic Pressure | 2.10 ± 0.90 | 2.11 ± 0.86 | 1.94 ± 0.83 | 2.25 ± 0.90 |
| Maladaptation | 1.72 ± 0.68 | 1.88 ± 0.72 | 1.63 ± 0.63 | 1.97 ± 0.74 |
| Emotional Instability | 2.11 ± 0.83 | 2.09 ± 0.79 | 1.92 ± 0.74 | 2.26 ± 0.83 |
| Psychological Imbalance | 1.63 ± 0.66 | 1.61 ± 0.62 | 1.48 ± 0.54 | 1.75 ± 0.69 |
| Overall Mental Health Average | 1.95 ± 0.72 | 1.90 ± 0.69 | 1.73 ± 0.62 | 2.10 ± 0.71 |

**Note:** MMHI-60 = Middle School Student Mental Health Inventory. Higher scores indicate poorer mental health status. Middle school corresponds to Grades 7–9, and high school corresponds to Grades 10–12. This supplementary table presents descriptive mean ± SD values for the MMHI-60 sub-factors by educational stage and learning modality. The corresponding main effects and interaction effects were tested using two-way ANOVA in the main text.
