# Supplementary material for: The impact of learning modalities and sleep quality on adolescent mental health: a cross-sectional study
Source: Front Pediatr. 2026 Jul 22;14:1854377. doi: 10.3389/fped.2026.1854377 (PMC13437675; doi:10.3389/fped.2026.1854377)
Supplement: Supplementary file 2 [file Table2.docx]

**Supplementary Table S2.** Distribution of Sleep Patterns and Subjective Sleep Quality

| **Sleep Variable** | **Frequency (n)** | **Percentage (%)** |
| --- | --- | --- |
| Bedtime |  |  |
| < 22:00 | 363 | 6.3 |
| 22:00 | 1594 | 27.5 |
| 23:00 | 2412 | 41.7 |
| ≥ 24:00 | 1418 | 24.5 |
| Wake-up Time |  |  |
| ≤ 5:00 | 1805 | 31.2 |
| 06:00 | 2762 | 47.7 |
| 07:00 | 1009 | 17.4 |
| ≥ 8:00 | 211 | 3.7 |
| Subjective Sleep Duration |  |  |
| ≤ 5 hours | 809 | 14 |
| 6 hours | 2062 | 35.6 |
| 7 hours | 1698 | 29.3 |
| ≥ 8 hours | 1218 | 21.1 |
| Subjective Sleep Quality |  |  |
| Very Good | 1557 | 26.9 |
| Fairly Good | 2526 | 43.6 |
| Fairly Bad | 1371 | 23.7 |
| Very Bad | 333 | 5.8 |
