# Supplementary material for: The impact of learning modalities and sleep quality on adolescent mental health: a cross-sectional study
Source: Front Pediatr. 2026 Jul 22;14:1854377. doi: 10.3389/fped.2026.1854377 (PMC13437675; doi:10.3389/fped.2026.1854377)
Supplement: Supplementary file 3 [file Table3.docx]

**Supplementary Table S3. Grade-specific subjective sleep quality scores and actual sleep duration**

| Grade | Subjective sleep quality (mean score, range 0–3, higher = poorer) | Actual sleep duration (mean hours per night) |
| --- | --- | --- |
| 7 | 1.2 | 7.4 |
| 8 | 1.8 | 7.1 |
| 9 | 1.4 | 6.9 |
| 10 | 1.3 | 6.4 |
| 11 | 1.5 | 6.1 |
| 12 | 1.6 | 5.9 |
